# Substitute services: a barrier to controlling long-term care expenditures

**DOI:** 10.1007/s10433-020-00570-x

**Published:** 2020-06-11

**Authors:** Mark Kattenberg, Pieter Bakx

**Affiliations:** 1 CPB Netherlands Bureau for Economic Policy Analysis, The Hague, The Netherlands; 2 Erasmus School of Health Policy and Management, Erasmus University Rotterdam, Rotterdam, The Netherlands

**Keywords:** Long-term care, Public insurance, Administrative data, Home care, Social care

## Abstract

In many developed countries, long-term care expenditures are a major source of concern, which has urged policy makers to reduce costs. However, long-term care financing is highly fragmented in most countries and hence reducing total costs might be complicated by spillover effects: spending reductions on one type of care may be offset elsewhere in the system if consumers shop around for substitutes. These spillovers may be substantial, as we show using a reform in the budget for municipalities for the most common type of publicly financed home care in the Netherlands, domestic help. This reform generated an exogenous change in the grant for domestic help that does not depend on changes in its demand. We show that the change in budget affected consumption of this care type, but that this effect was mitigated by offsetting changes in the consumption of three other types of home care that are financed through another public scheme and are organized through regional single payers. We find that a 10 euro increase in the grant for domestic help increased use of domestic help and nursing by 0.13 and 0.03 h per capita (4.4 and 5.2% of use in 2007), whereas it decreases use of individual assistance and personal care by 0.03 and 0.05 h per capita (4.1 and 2.9% of use in 2010 and 2007, respectively). As a result, the total spending effect is closer to zero than the effect on domestic help suggests. This finding means that the fragmentation of long-term care financing limits the ability to control expenditure growth.

## Introduction

Government intervention to ensure equal access to long-term care (LTC) and to limit financial risk is heavy, and hence most spending on LTC comes from the public purse, even in the USA (OECD 2018). Rising expenditures on long-term care for older people are a major concern in all OECD countries (OECD 2011). In the Netherlands, for example, rising LTC costs have been identified as the most important threat to the sustainability of overall public finance (CPB 2014). Furthermore, the financing and organization of LTC is highly fragmented in virtually all these countries, which means that curbing spending growth requires concerted action from a number of organizations, whose incentives may not be well-aligned with each other (OECD 2011; Bakx et al. 2015). Consequently, the appropriate amount and the appropriate mix of home care services may not be achieved. This may also have an effect on the use of other, more expensive types of care such as nursing home care and hospital care.

These spillover effects are a threat, in particular if types of LTC financed from different sources are substitutes.^1^ The effect is likely to be the largest for similar types of LTC, i.e., among types of home care, rather than between home care and institutional care, for instance. However, most of the previous studies have estimated substitution effects between broad classes of LTC—formal home care, informal home care and institutional care—and between LTC and other types of health care (Ettner 1994; Pezzin et al. 1996; McKnight 2006; Stabile et al. 2006; Bonsang 2009, Orsini 2010; Weissert and Frederick 2013; Guo et al. 2015; Karlsberg Schaffer 2015; Goncalves and Weaver 2017; Hollingsworth et al. 2017). These studies disregard substitution among different types of home care, possibly because survey data usually contain only a limited amount of detail on LTC use.^2^ Consequently, not much is known about the forces driving the composition of home care, despite the important role assigned to home care when it comes to the containment of rising health care costs.

One of these forces may be the that home care financing is often fragmented and that this fragmentation causes spillovers: spending reductions on one type of care may be offset elsewhere in the system if consumers shop around for substitutes. We study spillovers across five types of home care (domestic help, individual assistance, group assistance, nursing and personal care). Of these, domestic help is the most common type of home health care and is it is organized by municipalities. Hence, we study: did a reform in the grant to municipalities to organize domestic help in the Netherlands also affect the use of the other types of home care, which are not organized by municipalities and which are financed through another scheme? Such spillovers may occur if individuals who are not getting all the domestic help that they need request more of the other types of home care to compensate for this. Evidence of such effects would indicate that changing the level of LTC spending by changing subsidies on one type of care at a time may not be very effective.

To this end, we use administrative panel data from the Netherlands containing much more details about home care use than typical household surveys. We observe the use of LTC provided at home (domestic help, individual assistance, nursing and personal care) as well as the use of LTC provided outside the home environment (group assistance and institutional care). Moreover, we exploit that the magnitude of the reform in the subsidies for domestic help varied across the 400 municipalities which generates substantial variation within regions. These data have been used to study a number of related topics, including (correlations in) variation of home care use by older persons, e.g., across regions, or groups with different health problems or socioeconomic status (Algemene Rekenkamer 2015; CPB/SCP 2015; SCP 2017). But they have not been used yet for estimating spillovers.

## Home care in the Netherlands

In the Netherlands, virtually all LTC is publicly financed and organized. During the study period (2007–2013), this was done through two schemes.^3^ The first scheme was the Exceptional Medical Expense Act (EMEA), which provides universal and mandatory public LTC insurance, the second was the Social Support Act (SSA). The schemes were complementary with regard to the types of care they cover: Domestic help is paid for through the SSA and organized by municipalities, the other types of home care and institutional care are financed and organized through the EMEA.

In both schemes, the main way in which demand for care is restricted is through eligibility assessments. Individuals who want to use home care or institutional care put in a request for an eligibility assessment at either the independent agency responsible for the assessment for EMEA-financed care, at the municipality for SSA-financed care, or both. The contents of these assessments differ and they are carried out separately. The EMEA and SSA furthermore differ in the way the care is funded and providers are contracted, among other things. Table 1 provides a summary of the institutional characteristics of both schemes.

**Table 1:** Public LTC financing in the Netherlands. *Source*: CBS (2017), Zorgcijfers (2015)

	Public LTC insurance	Public provision of LTC
Legal basis	Exceptional Medical Expenditure Act	Social Support Act
Period	1968–2015^a^	2007–current
Home care benefits (2013 spending in billion euros)^b^	Nursing (0.447), personal care (2.144), individual assistance (0.730), group assistance (0.490)	Domestic help (1.612)
Scheme also pays for:	Institutional care, long-term mental health care, assistance and transportation	Social work, social policy, home adaptations
Funded through	Designated insurance premium (73%) general taxation (18%), cost sharing (9%)	Lump sum grant paid from general taxation (80%), cost sharing (20%)
Organizer	32 regional single payers	408 municipalities (in 2013)
Financial risk	National government sets binding ceiling for care expenditures	408 municipalities determine expenditures on care
Eligibility decisions	10 regional offices of an independent agency, based on national guidelines	408 municipalities, based on local guidelines (provided compensation for ADL problems is ‘adequate’)
Insured population	Universal	Universal

^a^Replaced by the Health Insurance Act (2006–current) and the new Long-Term Care Act. ^b^In-kind provision only

This split in LTC financing had been in place since 2007. Domestic help had been funded through the EMEA scheme but was made a responsibility of municipalities under the SSA in 2007. This change was intended to curb LTC expenditures (Tweede Kamer 2004) and municipalities indeed kept use of domestic help in check as was intended: it increased by only 1% per year between 2007 and 2013, while total LTC spending increased by 30% over the same period (CBS 2014).

A major reason for this was that municipalities were given the means and incentives to do so. The main way in which municipalities can reduce spending is through tightening the eligibility rules. Municipalities have considerable freedom in setting these rules as long as they adequately compensate inhabitants who cannot perform daily housekeeping activities on their own and who cannot rely on others in their network to do them. By law, municipalities do not have to provide domestic help if informal caregivers can assist the patient (Tweede Kamer 2004). In addition, municipalities had an incentive to cut down on spending. Municipalities are compensated by an unconditional block grant and spending it on other things is explicitly allowed (Department of the Interior 2007).

### The grant reform

The grant that municipalities received for domestic help in 2007 was based on expenditures in 2005. From 2008, onwards, the grant was calculated differently: a risk-adjustment formula has been used to determine the amount that each of the municipalities received. The formula makes use of information about the composition of the population and the need for care, which can arguably not be affected by the municipalities themselves or the regional single payers that organize the EMEA-financed care (see “Appendix” for details). The grant reform that we analyze entailed a change in the *weights* of the risk-adjustment formula that was implemented in 2011. Specifically, the revised version attached more weight to indicators on income and health care demand and supply (Kattenberg and Vermeulen 2017). This adjusted formula was announced in 2010 (Department of the Interior 2010) following claims by municipalities in the east and south of the country that the initial formula did not reflect demand for domestic help in their jurisdictions (see for instance Notenboom et al. 2008). This reform redistributed approximately 41 million euro among municipalities.

### Potential for spillovers from a reform

Older adults in the Netherlands and others who need help^4^ can use five types of home care^5^—domestic help, individual assistance, group assistance, personal care and nursing—which enable them to live at home despite their need for assistance. They often use multiple types of home care: 50% of the older adults using personal care or nursing also uses domestic help (Jonker et al. 2007).

Domestic help is the most common type of home care: about 40% of home care is provided as domestic help. Persons receiving domestic help are helped with cleaning their house, getting groceries or cooking. People with mental disabilities are also eligible for domestic help when they need help planning or organizing housekeeping activities. The latter form of domestic help can therefore overlap with (individual) assistance, a form of LTC in which patients receive *general* assistance in organizing their lives.^6^

Other types of LTC are personal care (i.e., assistance with daily activities like dressing, showering or assistance in eating) and nursing (i.e., nursing tasks like cleaning wounds or providing medicine) and group assistance (i.e., learning to perform daily activities despite their functional limitations). Of these, personal care is closely related to domestic help, although the tasks formally do not overlap, but are complementary. For instance, cooking and laying the table are domestic help while assistance with eating is personal care.

Because of these types of overlapping or complementary tasks, a change in the budget for domestic care may have spillover effects to personal care, nursing and individual assistance. This may occur when individuals who are eligible for fewer hours of domestic help than needed or wanted requests eligibility for other types of home care instead to make up for the deficit. Indeed, the majority of nurses who provide personal care, individual assistance or nursing report they sometimes perform housekeeping activities to lower the burden on informal caregivers when no immediate domestic help is available (Kuiken and Pronk, 2016). In addition, there may be an indirect effect of on the use of other types of home care because increased domestic care use may help to postpone a nursing home admission (cf. Guo et al. 2015) and thus increase rather than decrease the use of other types of home care.

## Data and methods

### Data

To study whether there are spillovers from domestic care to the other types of home care and to institutional care, we link administrative data at the municipal level; this is the level at which the reform of interest occurred. Information on the domestic help grant in 2007 and 2013 comes from the Department of the Interior (2007, 2014). From this information, we calculate the effect of the reform on the per capita grant that municipalities receive by rescaling the grant using pre-reform population estimates to remove the influence of population changes (see methods sub section and “Appendix” for details). This information is linked to data on the use of each of the types of home care (in hours per capita)^7^ and use of institutional care (in days per capita) in these years from administrative records from the Central Administration Office. Furthermore, we link population characteristics and data on election outcomes that are used as a proxy for local preferences from Statistics Netherlands and the Electoral Council (2014), respectively.^8^

## Methods

To find out whether spillover effects matter when changing spending on one type of home care, we investigate how the change in the weights of the risk-adjustment formula used for the financing of domestic help in 2011 affected the use of each of the LTC types. We do so by estimating Eq. (1) for each of the four types home care. In this equation, the change in use (Δ*h*_*i*_) is explained by the change in the grant for domestic help caused by the reform between the years 2007 and 2013 (Δ*G*_*i*_):

1 $$ \Delta h_{i} = C + \beta \Delta G_{i} + E_{r} + \varepsilon_{i} $$

To estimate this relationship, we need to account for third factors and general time trends. To deal with time-invariant differences at the municipal level, we take first differences between 2007 and 2013. To deal with time-variant differences, we proceed in three steps. First, the constant *C* controls for time-variant effects that are common to all municipalities, including national-level reforms.^9^ The EMEA-region specific effects *E*_*r*_ capture any deviation from this time trend at the EMEA-region level, which is the level at which regional single payers organize other types of home care. Second, to make sure the estimate on the grant is not biased by unobserved changes in demand for LTC, we follow Kattenberg and Vermeulen (2017) and only use the part of the change in the grant for domestic help caused by the reform, *ΔG*_*i*_ (“Appendix” contains a detailed description of the reform). We refer to this measure as the *counterfactual grant change.* The counterfactual grant change differs from the observed grant change as it only reflects the change in grant for domestic help that is due to the reform. Importantly, counterfactual grant change does not depend on changes in municipal characteristics that affect demand for health care, and therefore the omission of these characteristics from Eq. (1) does not bias the estimated parameter $\beta$.^10^

In short, we have constructed the counterfactual grant change as follows. The grant reform entailed a change in the *weights* of the risk-adjustment formula that is used to calculate the grant. This risk adjustment is based on indicators of need at the municipal level. Over time, however, the population composition of municipalities changes too and thus the *level* of each of the need indicators changes. As we are only interested in the effect of the reform (i.e., the change in the weights), we need to remove the effect of the population composition on the grant. Hence, we derive the counterfactual grant change *ΔG*_*i*_ in three steps. First, we calculate the size of the grant for domestic help that municipalities would have received in 2013 if their need indicators would have remained at their 2005 values. To this end, we multiply the weights of the need indicators in 2013 with the levels of these indicators from 2005. Second, we scale these amounts to the total amount of grant money received by municipalities in 2013. Third, we subtract the amount of grant money received in 2007, which are also based on information from 2005, to compute the difference over time. As a result, the variable *ΔG*_*i*_ only contains variation caused by the reform of the grant allocation and no variation caused by the change in local demand for LTC.

As explained, the use of the counterfactual grant limits the risk that the estimated parameter $\beta$ is biased due to omitted variable bias. Another advantage of analyzing the grant reform is that it did not affect the EMEA scheme directly. This simplifies the interpretation of the results: any effect on home care within the EMEA scheme that can be attributed to the grant reform should run via changes in municipal policies. Equation (1) is a reduced form estimate of the effect of the reform on the five types home care, which means we are agnostic about the adjustment processes driving the results.^11^ The result may not only be driven by changes in use of domestic help, but also by the use of other municipal social services and there may be direct and indirect effects (e.g., through informal care or other types of formal care). Although we cannot separately identify the drivers of adjustments, the results are still informative as reforms in these type of grants are a major policy instrument for national governments in shaping the mix of home care that older people receive, especially in the context of home care, which is often organized at the local or regional level and financed through a patchwork of schemes (Bakx et al. 2015; OECD 2011).

Finally, as the reform itself might be targeted toward specific municipalities,^12^ in a series of robustness checks, we include control variables for pre-reform demand for LTC and political preferences at the municipal level. Specifically, we include control variables on age, income and the health condition of the municipality population as these variables might affect demand for home care. We also include the share of the population belonging to a minority group and population density as minorities make less use of domestic help and providing domestic help is less expensive in densely populated areas (due to reduced travelling time), see Jonker et al. (2007) and Van Eijkel et al. (2017). Furthermore, we condition on the main intergovernmental (block) grant that municipalities receive from the central government and the vote shares going to (1) left wing parties, (2) Christian democratic parties and (3) local and other parties. We do so as the budget and the composition of the municipal council might affect the budget the municipality spends on health care and social services. We condition for these demand and supply characteristics by including lagged levels of these covariates, i.e., they are measured before the reform took place. In addition, we perform the robustness checks by controlling for the difference in these covariates over the period 2007–2013.^13^

## Results

### Descriptive statistics

**Fig. 1:**
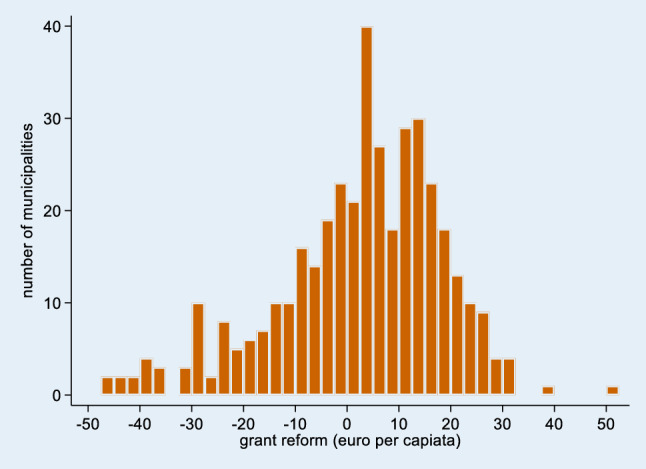
Distribution of reform in grant for domestic help (2007–2013)

Table 2 shows that the average per capita grant for domestic help rose by 3.48 euro per capita (3.8%). Part of this increase is caused by changes in the composition of the population, as illustrated by the lower average counterfactual grant change of (2.48 euro per capita on average). This average amount masks substantial changes at the municipal level, however, as for some municipalities the value of the counterfactual grant changed by more than 40 euro per capita (Fig. 1).

**Table 2:** Descriptive statistics

	Mean	SD	Minimum	Maximum	*N*
*LTC use per capita in minutes: change over time*					
Domestic help	9.0	37.2	− 201.6	109.2	400
Personal care	54.6	33.6	− 34.8	186.0	400
Nursing	− 15.6	10.8	− 72.6	23.4	400
Individual assistance (2010–2013)	0.0	13.8	− 93.0	51.6	394
Group assistance (2010–2013)	6.0	12.0	− 33.6	61.8	394
Institutional care	− 6.0	42.6	− 240.0	228.6	400
*Domestic help grant (euros per capita): change over time*					
Change grant for domestic help 2007–2013	3.48	18.06	− 55.12	61.15	400
Reform of the grant for domestic help	2.58	15.97	− 47.10	52.28	400
*Lagged control variables*					
Vote share left wing parties	26.83	15.04	0.00	73.75	400
Vote share Christian democratic parties	26.75	13.16	0.00	86.92	400
Vote share local and other parties	29.97	17.63	0.00	100.00	400
Share of the population aged 75 or older	6.29	1.53	2.61	13.16	400
Average personal income in 1.000 euro	14.60	1.59	10.21	23.06	400
Share of the population belonging to a minority group	12.38	7.16	2.29	50.89	400
Mortality rate	0.82	0.19	0.31	1.87	400
Population density	763.56	931.59	25.00	5711.00	400
*Change in control variables*					
Vote share left wing parties	− 7.82	8.36	− 60.01	17.56	400
Vote share Christian democratic parties	− 3.90	8.85	− 62.61	23.33	400
Vote share local and other parties	7.73	19.82	− 27.19	99.30	400
Share of the population aged 75 or older	1.09	0.59	− 0.81	3.01	400
Average personal income	− 1.33	0.86	− 9.06	2.93	400
Share of the population belonging to a minority group	0.27	0.38	− 0.53	2.88	400
Mortality rate	0.07	0.11	− 0.25	0.61	400
Population density	12.42	73.24	− 471.35	430.00	400

Changes in LTC use are for the period 2007–2013, unless specified otherwise. Assistance in 2010 observed from week 25 onwards. Therefore, the change in uptake of individual assistance and group assistance between 2010 and 2013 is computed after multiplying the 2010 observations with 1/(52–25)*52. Domestic help, individual assistance, nursing and personal care are measured in total hours divided by the municipal population in 2000, group assistance and institutional care are measured in total shifts and total days divided by the population in 2000, respectively. Lagged control variables are measured in 2005, except for the vote shares, which are the outcome of the 2006 election. Change in control variables are the change over the period 2007–2013, except for the vote share, which are the change between 2006 and 2010

Trends in use differ by care type. Use of domestic help rose moderately on average, whereas personal care use and group assistance increased strongly and use of nursing fell substantially. The use of individual assistance was virtually unchanged.

### Regression results

**Fig. 2:**
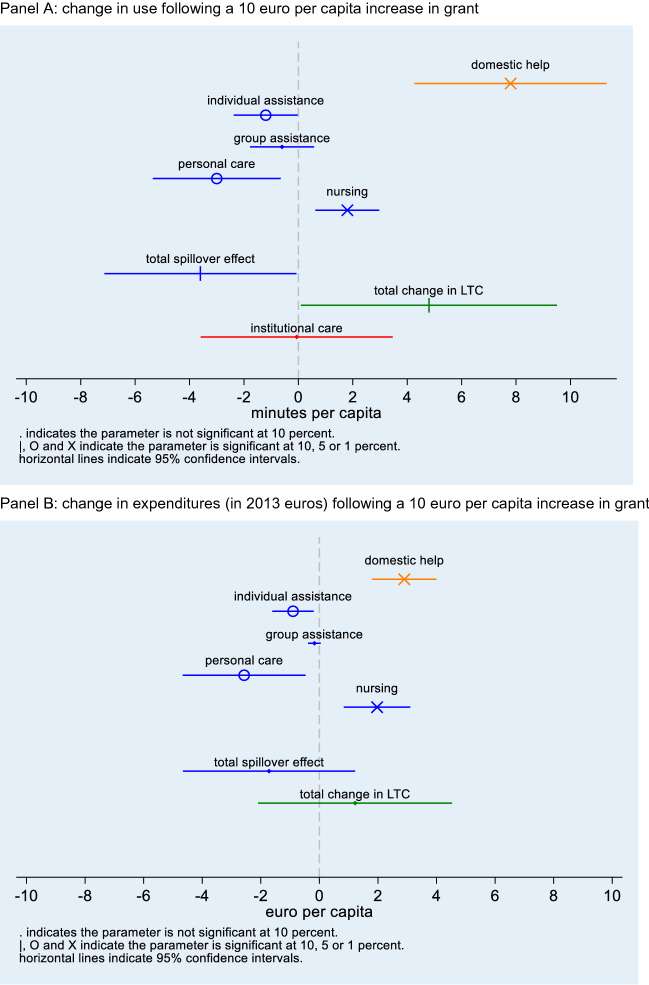
Illustration of main regression results in Table 3. Panel **a**: change in use following a 10 euro per capita increase in grant. Panel **b**: change in expenditures (in 2013 euros) following a 10 euro per capita increase in grant

Table 3 and Fig. 2 summarize our main regression results. They suggest a ten-euro increase in the grant for domestic help increases the use of domestic help with 0.13 h, i.e., 8 min (column 1 of Table 3). At an average rate of 22 euro per hour (Van Eijkel et al. 2017—Appendix Table 5), this means that municipalities spend about 3 euro on this specific service when the grant increases by 10 euro (column 2 of Table 3).

**Table 3:** Regression results

Dependent variable	(1)	(2)	*N*
	Effect of grant reform on per capita use in hours	Effect of grant reform on per capita spending in 2013 euros	
Δ Domestic help	0.013 (0.003)***	€0.290 (€0.056)***	400
*EMEA*-*financed care*			
Total spillover effect	− 0.006 (0.003)*	− €0.172 (€0.150)	394
Δ Personal care	− 0.005 (0.002)**	− €0.257 (€0.107)**	400
Δ Nursing	0.003 (0.001)***	€0.197 (€0.058)***	400
Δ Assistance (group)	− 0.001 (0.001)	− €0.017 (€0.011)	394
Δ Assistance (individual)	− 0.002 (0.001)**	− €0.090 (€0.036)**	394
*All home care*			
Total change	0.008 (0.004)*	€0.122 (€0.169)	394
*Nursing home care*			
Δ Institutional care	− 0.000 (0.003)	–	400

Dependent variables measured in total hours divided by municipal population in 2000. The grant reform is measured as total euro divided by municipal population in 2000. Changes in dependent and independent variables are calculated as the difference between 2013 and 2007 values, except for the analyses with the change in the use of assistance as the dependent variable for which the difference is between 2013 and 2010. Expenditures on LTC computed by multiplying use of care times prices of LTC (listed in Table 5). All specifications contain indicators for EMEA regions. Robust standard errors in parentheses. * *p* < 0.1, ** *p* < 0.05, *** *p* < 0.01

Next, we consider the effect on overall EMEA-financed home care. Our results present some evidence that a ten-euro per capita increase in the grant for domestic help causes a drop in EMEA-financed home care of 4 min per capita (ten times 0.06 h), but it had no significant effect on total expenditures on these types of care. This difference occurs because the prices of EMEA-financed types of care differ.

Specifically, Table 3 shows that a ten-euro increase in the grant for domestic help leads to a decrease in the use of personal care of 3 min per capita (ten times 0.005 h times 60 min) and of individual assistance of 1 min per capita (ten times 0.002 h times 60 min). Using listed maximum prices of these types of home care, these changes convert to a 2.6 euro decrease in expenditures on personal care and a 0.9 euro decrease in expenditures on assistance. For nursing we find evidence suggesting the opposite: a ten euro increase in the grant for domestic help increases the use of nursing by 2 min per capita implying a 2.0 euro increase in expenditures.^14^ We do not find evidence that the use or expenditures on group assistance are affected by the reform in the grant for domestic help (Table 3). As group assistance is related to the other types of LTC, yet provided outside of the home environment, this strengthens our belief that our estimates reflect substitution of LTC provided at home.

Taken together, the effects of the reform of the grant for domestic help on i) the use of domestic help and ii) on EMEA-financed home care show that a ten-euro increase in the grant for domestic help increases the use of home care by about 5 min (ten times 0.008 h times 60 min) per capita. This estimate is almost 40% lower than the effect of the reform on domestic help alone. In fact, the change in aggregate home care spending, which is the sum of the changes in the use of each of the types of home care multiplied by their respective prices, is not significantly different from zero. Finally, we do not find evidence that the reform in the grant for domestic help influenced institutional care use.

The robustness checks show that the results are largely unaffected after including four sets of lagged level or changes of variables that proxy for preferences for LTC policy (Table 4—Columns 2 and 3), proxies for LTC demand (Columns 4 and 5), changes in the other funds that municipalities receive from the central government (Column 6) and changes in the hourly co-payment for domestic help (Column 7). The effects on individual types of home care are the same or similar in all specifications, while the total spillover effect is significant at the 10-percent level in all but two specifications. The effect on the total number of hours of home care used is significant and equal in all specifications.

**Table 4:** Robustness checks

Additional covariates included	(1)	(2)	(3)	(4)	(5)	(6)	(7)	(8)
	Main specification	Initial vote shares	Change in vote share	Initial population composition	Change in population composition	Change in total grant	Change in domestic help co-payment	*N*
*Dependent variables (in hours)*								
Δ Domestic help	0.013 (0.003)***	0.013 (0.003)***	0.013 (0.003)***	0.013 (0.003)***	0.013 (0.003)***	0.013 (0.003)***	0.013 (0.002)***	400
*EMEA*-*financed care*								
Total spillover effect	0.008 (0.004)*	0.008 (0.004)*	0.008 (0.004)*	0.007 (0.004)	0.008 (0.004)*	0.007 (0.004)	0.007 (0.004)*	394
Δ Personal care	− 0.005 (0.002)**	− 0.005 (0.002)**	− 0.005 (0.002)**	− 0.006 (0.002)***	− 0.005 (0.002)**	− 0.006 (0.002)**	− 0.005 (0.002)**	400
Δ Nursing	0.003 (0.001)***	0.003 (0.001)***	0.003 (0.001)***	0.003 (0.001)***	0.002 (0.001)***	0.003 (0.001)***	0.003 (0.001)***	400
Δ Assistance (group)	− 0.001 (0.001)	− 0.002 (0.001)*	− 0.001 (0.001)	− 0.001 (0.001)	− 0.002 (0.001)*	− 0.001 (0.001)	− 0.001 (0.001)	394
Δ Assistance (individual)	− 0.002 (0.001)**	− 0.002 (0.001)**	− 0.002 (0.001)**	− 0.002 (0.001)**	− 0.001 (0.001)*	− 0.002 (0.001)**	− 0.002 (0.001)**	394
*All home care*								
Total change	− 0.006 (0.003)*	− 0.006 (0.003)*	− 0.006 (0.003)*	− 0.006 (0.003)**	− 0.005 (0.003)*	− 0.006 (0.003)**	− 0.005 (0.003)*	394
*Nursing home care (in days)*								
Δ Institutional care	− 0.000 (0.003)	− 0.001 (0.003)	− 0.000 (0.003)	− 0.002 (0.003)	− 0.002 (0.003)	− 0.001 (0.003)	− 0.000 (0.003)	400

Changes in dependent and independent variables are calculated as the difference between 2013 and 2007 values, except for the analyses with the change in the use of assistance as the dependent variable for which the difference is between 2013 and 2010. All specifications contain indicators for EMEA regions. Robust standard errors in parentheses

*p < 0.1; **p < 0.05; ***p < 0.01

## Conclusion

In most countries, LTC is subsidized and organized through a patchwork of public schemes. Changes in one scheme have implications for use of the care subsidized through other schemes and these spillover effects need to be accounted for when evaluating the effects of a reform. This article exploits detailed administrative records on LTC use and exogenous changes in the grant that municipalities in the Netherlands receive to organize domestic help to estimate the spillover effects on other types of home care and on institutional care which are financed through another financing scheme.

Prior research (Ettner 1994; Pezzin et al. 1996; McKnight 2006; Stabile et al. 2006; Stuck 2008; Bonsang 2009, Orsini 2010; Weissert and Frederick 2013; Guo et al. 2015; Karlsberg Schaffer 2015; Goncalves and Weaver 2017; Hollingsworth et al. 2017) has treated home care as a single type of care, possibly because of data limitations, and focused on substitution of home care with institutional care and informal care. Yet, substitution between different types of home care financed through separate systems is at least as likely, and our results show that it might be as relevant for governments and insurers seeking to limit public spending and ensuring an effective and equitable allocation.

The reform in the grant for domestic help increased the use of domestic help: A 10 euro increase led to an 8-minute increase on average meaning that municipalities spent on average 30% of the additional funds on domestic help. In addition, the results show that there are substantial spillovers from the reform: A 10 euro increase in the grant decreased the use of other types of home care targeted at older people by almost 4 min. Consequently, almost half of the change in domestic help use is undone by the changes in the other types of home care. This spillover effect means that the reform in the grant for domestic help had a smaller effect on total home care use than is apparent from studying the change in domestic help use alone. In monetary terms, the spillover effects on the other, more expensive, types of home care are even more striking: The change in spending on domestic help is cancelled out by decreases on the other types of LTC provided at home, and we cannot reject the null hypothesis that aggregate LTC expenditures remained unaffected.

Like McKnight (2006) but unlike a couple of other studies (Ettner 1994; Pezzin et al. 1996; Orsini 2010; Guo et al. 2015), we do not find an effect of changes in home care subsidies on use of institutional care. A potential explanation for the absence of an effect on institutional care is that formal care delivered at home or informal caregiving is a closer substitute than institutional care for domestic help.

Our results are of direct relevance to policymakers in the Netherlands as well as in other countries in which the financing of LTC is fragmented. As Belgium and Switzerland have the same split in home care funding as the Netherlands (Bakx et al. 2015)—and in many other countries LTC financing is split between separate schemes in other ways (OECD 2011)—these findings suggest that similar spillover effects might occur elsewhere. The limited ability to alter long-term care spending growth caused by spillover effects that we document is likely to be an inherent negative consequence of this fragmentation.

A major strength of our analysis is that we use detailed administrative data on all home care use in the Netherlands and that the level of an observation in this data is also the level at which decisions are made. Moreover, our analysis limits the omitted variable bias and the main variable of interest—the change in the grant to municipalities—is also the main policy lever that is used to influence the use of domestic help, which facilitates a straightforward interpretation of the result and enhances its relevance. A limitation of our analysis is that municipalities are free to spend to grant as they wish and that the change in the grant may have affected other programs, which in turn affects the use of home care as well. There is no data available to estimate the relative importance of these other channels and consequently we only estimate the total effect of the grant change on the use of home care, not how this total effect came about. Moreover, reforms that affect the amount and the composition of home care that is provided might also influence the amount of informal care that is provided. Because there is no data at the municipal level about informal caregiving in the Netherlands, we have not studied this spillover, but prior research from other countries shows that this might indeed occur (Stabile et al. 2006; Karlsberg Schaffer 2015).

Moreover, in 2015, a broad set of LTC reforms has been implemented in the Netherlands which further complicated LTC financing. These reforms included a 30% reduction in the budget that municipalities received for domestic help (Department of the Interior 2014) and the decentralization of assistance to municipalities. The other types of home care are now financed through the Health Insurance Act and organized by health insurers, while institutional care is still covered through the public LTC insurance scheme. Although the results of our study cannot be translated one-to-one to this new situation, our results do suggest that these reforms may urge municipalities to integrate assistance, domestic help and other services that they offer. This is an advantage, because our results show that there are substantial spillovers to the use assistance when the budget for domestic help changes and municipalities are now incentivized and thus more likely to incorporate these spillovers on assistance when deciding about spending on domestic help. On the other hand, these reforms mean that coordination between assistance and the types of home care that are now organized by health insurers (nursing and personal care) has become more complicated. If similar spillovers exist when the budget for assistance is changed as we observed for domestic help, this means that budget cuts on assistance might lead to higher costs for health insurers.

Furthermore, our results suggest that these spillover effects matter for public LTC financing: The effect of changes in the budget for domestic help on total home care spending was mitigated because they are compensated for elsewhere in the system. Hence, our results lend credibility to the belief that the 2015 budget cuts had important spillovers to other types of home care, but not to institutional care or group assistance. Moreover, our results show that a full evaluation of reforms of the effects complicated LTC financing systems in the Netherlands and elsewhere should focus on all types of home care, including those financed through other systems. In addition, our results show that decentralization by providing a block grant to municipalities means that a large part of the funds may be spent on other things than home care when the funds are not earmarked.

Lastly, although it can be optimal to finance related health care services in separate schemes, our results show that such a split can reduce the ability to keep total spending in check because of coordination problems. As LTC financing is split between separate schemes in many countries (OECD 2011; Bakx et al. 2015), these findings suggest that similar spillover effects might occur elsewhere too. These spillovers need to be considered when reforming LTC financing. In addition, societies that seek to limit LTC spending in the long run may consider alternative ways of achieving this, including reablement programs that limit the development functional limitations or restore abilities in older populations (cf. Lewin et al. 2013; Tuntland et al. 2015; Winkel et al. 2015; Zingmark et al. 2017, 2019; Metzelthin et al. 2018).

## Appendix

### Types of LTC

Table 5 describes the types of LTC studied in this paper.

**Table 5:** Types of LTC

Type	Price	Brief description
Domestic help	23	Help with managing the household
Group assistance	46.82	Assistance with maintaining a structured life by providing group activities
Individual assistance	53.29	Assistance with activities of daily living and with maintaining a structured life; increasing self-reliance (including psychosocial self-reliance)
Personal care	49.45	Performing tasks that a person usually carries out herself (self-care)
Nursing	73.88	Performing nursing tasks; signaling, supporting and counseling; practicing self-care

Descriptions based on CIZ (2012), van Van Eijkel et al. 2017). Prices are maximum unit prices in euro in 2013, except for domestic help which is the average price per hour (Van Eijkel et al. 2017; NZA 2013a, b)

### Description of the domestic help grant allocation

This appendix provides more detail on the grant allocation formula. The formula was developed for the Department of the Interior and was used for the first time in 2008. A revised version, implemented in 2011, attached more weight to indicators on income and health care demand and supply (see Kattenberg and Vermeulen 2017). In Table 6, we list the 23 need indicators and the weights that were used to distribute the grant for domestic help to municipalities in 2013 (column 1). The size of the grant a municipality received in 2013 equaled the sum of the municipal score on the need-indicators times their weights.

Column 2 shows the average share of grant money distributed by a need-indicator. Clearly, not all need-indicators are equally important: the most important variable distributes about 20% of grant money on average, whereas the least important one distributes less than 0.1 promille. We have ordered the need-indicators into four groups. This grouping shows that about two-third of the grant is distributed using eight indicators related to average income. Almost 20% of grant money is distributed using eleven variables on municipal population and about 12% is distributed using two indicators on health care demand and supply. The fourth group of other indicators distributed only about 1%.

The change in the grant for domestic help due to the reform is computed as follows: First, we take the 2013 weights of the allocation formula and multiply them with the corresponding demand indicators measured in 2005. We use this result to compute the share of total grant money that each municipality would have received in 2013 given the value of the demand indicators in 2005. Second, from this share we subtract the share of total grant money a municipality received in 2007. Third, this difference is multiplied by the total amount of grant money supplied in 2007 and divided by population of the municipality to express the grant change in euro per capita. Defining the instrument this way means that variation is only caused by pre-reform needs.

**Table 6:** Allocation formula for domestic help in 2013

Indicator	Weight	Percentage^a^
*Indicators on composition of the municipal population*		
Population size	0.32	0.42
Population younger than 19	0.26	0.08
Population younger than 65	8.17	8.92
Population aged 65 or older and younger than 75	0.23	0.03
Population aged 75 or older and younger than 85	0.23	0.01
Population 85 or older	0.23	0.00
Population belonging to a minority group	0.83	0.09
Potential number of people visiting from nearby municipalities	1.49	1.96
Single person households with head aged 65 or older and younger than 75	31.77	0.92
Single person households with head aged 75 or older and younger than 85	127.06	3.38
Single person households with head aged 85 or older	222.36	2.92
Subtotal		18.7
*Indicators related to average income*		
Households with low income	0.98	0.17
Number of people who receive social support excluding those on welfare	80.36	5.82
Function of people with low incomes times the number of households with head aged 65–74^b^	263.20	3.93
Function of people with low incomes times the number of households with head aged 75–84^b^	1052.82	9.73
Function of people with low incomes times the number of households with head aged 85 or older^b^	1842.43	6.1
Function of relative average income times the number of households with head aged 65–74^c^	226.90	8.31
Function of relative average income times the number of households with head aged 75–84^c^	907.61	20.5
Function of relative average income times the number of households with head aged 85 or older^c^	1588.31	12.71
Subtotal		67.3
*Indicators on health care demand and supply*		
Local capacity health care^d^	1.01	1.80
Function of number of people who are chronically ill^e^	239.60	10.92
Subtotal		12.7
*Other indicators*		
Municipal housing density times housing stock over 1000	− 0.44	0.51
Lump sum transfer	24,263.31	0.78
Subtotal		1.3

^a^Share equal to the average value of the indicator times its weight divided by the sum of average values of indicators times their weights. As one weight is negative, we have used the absolute values of weights

^b^Function equal to the maximum of zero or [(the number of people with low income divided by the housing stock) minus 0.1]

^c^Function equal to the average municipal income over municipal income minus 0.55

^d^Equal to 26 times the capacity in mental health care plus 132.3 times the capacity in nursing houses plus 365 times the capacity mentally disabled health care. Capacity measured in number of beds

^e^Function equal to (share of population that is chronically ill minus 0.11) times population size

## Footnotes

1 Cross-price elasticities of demand in other health care settings have been studied extensively since the 1970s (e.g., Hill and Veney 1970; Davis and Russell 1972; Helms et al. 1978). Recently, most studies are about prescription drugs, see Goldman et al. (2007) and Glazer and McGuire (2012) for overviews of this strand of the literature.

2 Bonsang (2009) does however show that informal care differentially affects different types of home care: It is a complement to nursing but a substitute for domestic help.

3 Since 2015, LTC has been organized and financed through three schemes: the Long-Term Care Act, the Health Insurance Act and the SSA (CPB/SCP 2015). In the discussion we elaborate on the relevance of our results in the new financing scheme.

4 Neither of the schemes is exclusively for older people, yet 67% of all LTC spending is on users aged 65 and over.

5 Group assistance is not provided in the home of the care recipient and hence is, strictly speaking, not home care. Nonetheless, we include it here because its goal is closely related to the other types of home care and because it is funded and organized in the same way.

6 This overlap sometimes makes it difficult to distinguish whether someone should be eligible for domestic help or for individual assistance (CIZ 2010).

7 Except for group assistance, which is measured in the number of 4-h shifts. Use of domestic help is observed in 2007, 2010 and 2013, whereas use of assistance is observed in the last 6 months of 2010 and over 2013. Other types of health care are observed in 2007 and 2013. Eight municipalities granted inhabitants the right to a clean house and therefore hours of domestic help are not recorded by CAO. These municipalities are dropped and therefore we have data on 400 out of the 408 municipalities.

8 Municipalities follow four-year electoral cycles; we use data from the 2006 and 2010 elections.

9 All other health care is organized at the national level.

10 See the discussion of reduced form equation in chapter 4 in Angrist and Pischke (2008).

11 It would be tempting to use the grant reform as an instrument for use of domestic help in an instrumental variable analysis. However, the exclusion restriction for such a specification does not hold as municipalities are not obliged to spend additional funds on domestic help and they may spend part of the money on other services for frail older people.

12 For instance, Knight (2002) shows that the US federal government grants for highways is biased towards states with a large demand for highways.

13 These changes may be influenced by the reforms, making them bad controls, and hence are not included in the main specification (cf. Angrist and Pischke 2008).

14 The increase in nursing associated with the increase in the grant for domestic help may have many reasons. We speculate that it might for instance be that domestic help enables patients with severe functional limitations to leave the hospital sooner and that these patients need more nursing, or that increase domestic help enables the regional authorities that organize EMEA-financed care to restructure the home care that they provide. Highly specific data—or qualitative research—may shed light on which mechanisms may play a role. However, the distinction between nursing and personal care is abolished after the 2015 reform, meaning that the aggregate spillover effect matters most for policymakers.
